# A Simple Approach to Control the Physical and Chemical Features of Custom-Synthesized N-Doped Carbon Nanotubes and the Extent of Their Network Formation in Polymers: The Importance of Catalyst to Substrate Ratio

**DOI:** 10.3390/polym13234156

**Published:** 2021-11-27

**Authors:** Elnaz Erfanian, Milad Kamkar, Shital Patangrao Pawar, Yalda Zamani Keteklahijani, Mohammad Arjmand, Uttandaraman Sundararaj

**Affiliations:** 1 Department of Chemical and Petroleum Engineering, University of Calgary, Calgary, AB T2N 1N4, Canada; elnaz.erfanian@ucalgary.ca (E.E.); shital.pawar@ucalgary.ca (S.P.P.); yalda.zamaniketeklah@ucalgary.ca (Y.Z.K.); 2 School of Engineering, University of British Columbia, Kelowna, BC V1V 1V7, Canada; milad.kamkar@ubc.ca (M.K.); mohammad.arjmand@ubc.ca (M.A.)

**Keywords:** nitrogen-doped carbon nanotubes, electrical conductivity, rheology, conductive polymer nanocomposite

## Abstract

This study intends to reveal the significance of the catalyst to substrate ratio (C/S) on the structural and electrical features of the carbon nanotubes and their polymeric nanocomposites. Here, nitrogen-doped carbon nanotube (N-MWNT) was synthesized via a chemical vapor deposition (CVD) method using three ratios (by weight) of iron (Fe) catalyst to aluminum oxide (Al_2_O_3_) substrate, i.e.,1/9, 1/4, and 2/3, by changing the Fe concentration, i.e., 10, 20, and 40 wt.% Fe. Therefore, the synthesized N-MWNT are labelled as (N-MWNTs)_10_, (N-MWNTs)_20_, and (N-MWNTs)_40_. TEM, XPS, Raman spectroscopy, and TGA characterizations revealed that C/S ratio has a significant impact on the physical and chemical properties of the nanotubes. For instance, by increasing the Fe catalyst from 10 to 40 wt.%, carbon purity increased from 60 to 90 wt.% and the length of the nanotubes increased from 1.2 to 2.6 µm. Interestingly, regarding nanotube morphology, at the highest C/S ratio, the N-MWNTs displayed an open-channel structure, while at the lowest catalyst concentration the nanotubes featured a bamboo-like structure. Afterwards, the network characteristics of the N-MWNTs in a polyvinylidene fluoride (PVDF) matrix were studied using imaging techniques, AC electrical conductivity, and linear and nonlinear rheological measurements. The nanocomposites were prepared via a melt-mixing method at various loadings of the synthesized N-MWNTs. The rheological results confirmed that (N-MWNTs)_10_, at 0.5–2.0 wt.%, did not form any substantial network through the PVDF matrix, thereby exhibiting an electrically insulative behavior, even at a higher concentration of 3.0 wt.%. Although the optical microscopy, TEM, and rheological results confirmed that both (N-MWNTs)_20_ and (N-MWNTs)_40_ established a continuous 3D network within the PVDF matrix, (N-MWNTs)_40_/PVDF nanocomposites exhibited approximately one order of magnitude higher electrical conductivity. The higher electrical conductivity of (N-MWNTs)_40_/PVDF nanocomposites is attributed to the intrinsic chemical features of (N-MWNTs)_40_, such as nitrogen content and nitrogen bonding types.

## 1. Introduction

In the boom of the electronics industry, multifunctional materials have gained a great deal of interest from academia and industry. Multifunctional materials such as polymeric nanocomposites embedded with carbonaceous conductive nanofillers have been proposed as a versatile candidate for high tech electronics due to their excellent processability, strong corrosion-resistance, lightweight, flexibility, and tunable electrical properties, and low production cost compared to the conventional metal-based conductive materials [1]. Among various carbonaceous conductive fillers, carbon nanotubes (CNTs) depict enhanced electrical conductivity at a relatively small loading, owing to their low density, large specific surface, and high conductivity [2,3].

The final properties (e.g., electrical and rheological) of the CNT-based polymer nanocomposites are mainly controlled by inherent properties of CNTs (e.g., physical and chemical features) [4,5]. For instance, in terms of physical features, it was observed that high aspect ratio conductive fillers lead to an enhanced degree of interconnected networks, resulting in a higher electrical conductivity and rheological properties [6,7,8]. Arjmand et al. [9] showed that morphological features, such as the shape of the nanotubes, is closely linked with other parameters such as diameter, length, and synthesis yield of synthesized CNTs, and greatly affect electrical conductivity of the final product. Besides, large efforts have been involved in manipulation of the chemical properties of CNTs to design composites for diverse electrical applications. For instance, surfactant treated CNTs facilitated electrical conductivity of epoxy nanocomposites. Introduced surfactant on the CNTs surfaces acted as a bridge between CNTs and epoxy matrix [10]. Similarly, oxidized CNTs showed better dispersion quality and, thus, enhanced electrical conductivity in polyaniline nanocomposites [11].

In line with the demand for miniaturization [12,13] and optimization in the electronics industry, instead of conventional strategies, such as coating and surface modification of CNTs [14,15] or the use of hybrid additives [16,17], tuning specific intrinsic properties of CNTs in synthesis procedure seems to be a more effective and viable method to achieve the desired properties, meeting the requirements of advanced electrical applications. By altering synthesis parameters, such as temperature [18] to CNT substrates [19], and also by doping with different heteroatoms (nitrogen, boron, phosphorus, so forth), CNTs with different properties can be acquired. In this regard, nitrogen doping during synthesis was found to be an effective method to tune the intrinsic properties of CNTs [20]. Generally, the introduction of N atoms into carbonaceous materials can modulate electronic structures, which significantly impacts the electrical conductivity [21,22,23]. In this method, due to the different nitrogen content and various types of nitrogen bonding, i.e., graphitic, pyridinic, pyrrolic, and quaternary, physical and chemical features of the nitrogen-doped CNTs (N-MWNTs) can be controlled [20,24,25,26,27,28,29,30,31]. For instance, Liu et al. [32] showed that nitrogen concentration dictates the morphology of nanotubes to be bamboo-like, straight nanotubes, or inter-linked corrugated morphology.

In our previous studies, physical and chemical features of the CNTs were altered by changing the precursor gas ratio during the synthesis process [33], catalyst type [9,26], or synthesis temperature [4,34]. Despite a large set of studies performed to alter the electrical properties of CNTs, the effect of catalyst/substrate ratio is yet to be understood. In this regard, here, we successfully tuned the structural and electrical properties of the N-MWNTs via changing the catalyst/substrate ratio in a chemical vapor deposition (CVD) method. Taken together, considering the reports mentioning the effect of synthesis parameters on CNT properties, rather than changing the temperature, type of catalyst, synthesis time, or incorporating a secondary material, herein, we demonstrated that, with a simple factor (i.e., catalyst to substrate ratio), all the physical (e.g., length, diameter, and morphology) and chemical (e.g., N-doping content and type) features can be tuned. This study completes the understanding of the N-MWNTs synthesis and connects the broken link between various N-MWNT synthesis parameters and the resulting structural and electrical properties of N-MWNTs and their polymer-based nanocomposites in the literature.

## 2. Materials and Methods

### 2.1. Materials Synthesis

**Scheme 1:**
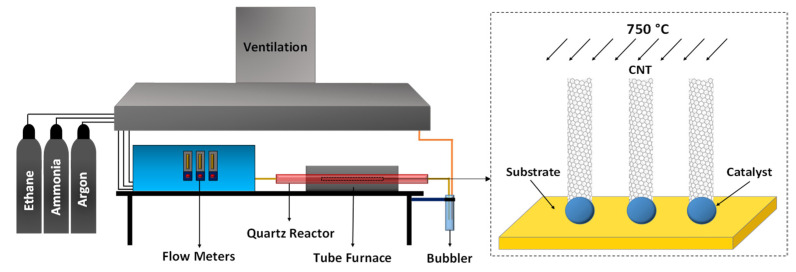
Graphic of CVD experimental setup.

Initially, the catalysts for the synthesis of nitrogen-doped CNTs (N-MWNTs) were prepared by incipient wetness impregnation of precursor dissolved in distilled water on aluminum oxide support (Sasol Catalox Sba-200). Then, the prepared catalyst underwent the drying and calcination procedures, and the reduction of catalyst particles was performed. The iron catalyst is one of the most popular catalysts for CNT synthesis due to its ability for easy decomposition of hydrocarbons, the high solubility of decomposed carbon in this metal at high temperatures, and high carbon diffusion rate in it [29,30]. Accordingly, the catalyst precursor used for N-MWNTs synthesis was iron (III) nitrate nanohydrate (Baker Analyzed ACS grade, Canada). aluminum oxide was also selected as the catalyst support owing to its highly porous structure that allows a strong support–metal interaction, which results in high metal dispersion and high density of active sites for the catalyst [29]. The presence of such strong interactions prevents the agglomeration of metal particles, which otherwise results in synthesis of graphitic particles and highly defective CNTs. The iron catalyst to alumina support loading was changed at three different concentrations: 10, 20, and 40 wt.% iron. The catalyst precursors were dried at ambient temperature for 24 h and 50 °C for 2 h. The catalyst calcination, reduction, and N-MWNTs synthesis were carried out in the chemical vapor deposition (CVD) setup. As shown in Scheme 1, the CVD setup has a quartz tubular reactor with an inner diameter of 4.5 cm enclosed within a furnace equipped with a heater. Two bubblers were used to absorb the gases created within N-MWNT synthesis. For the sake of conversion of the nitrate precursor into oxide precursor, the catalyst calcination was carried out at 350 °C under air atmosphere with a flow rate of 100 sccm for 4 h. Thereafter, the catalyst precursor was ground and sieved to achieve a fine powder. Then, it was reduced with hydrogen gas at a flow rate of 100 sccm and 400 °C for 1 h to obtain the alumina-supported iron catalyst for synthesis of N-MWNTs. N-MWNTs were then synthesized by passing a mixture of ethane, ammonia, and argon over the prepared catalysts. Ethane was the source of carbon, and ammonia and argon had the role of nitrogen source and inert gas carrier, respectively. The synthesis temperature, synthesis time, catalyst and substrate mass, and gas flow rate were set at 750 °C, 2 h, 0.6 g, and 150 sccm, respectively. Further information about the catalyst preparation procedure is detailed elsewhere [23,31]. A polyvinylidene fluoride (PVDF) matrix was purchased from 3M Canada (Grade: 11008/0001), with a melting point of 160 °C. Before mixing, the raw materials were dried in a vacuum oven at 60 °C overnight. Synthesized N-MWNTs were melt-mixed with the PVDF matrix at 210 °C and 235 rpm by using an APAM (Alberta Polymer Asymmetric Minimixer). The PVDF matrix was first masticated for 3 min, and then N-MWNTs were added into the mixing cup and mixed for an extra 14 min. The nanocomposites were prepared at different N-MWNTs loadings, i.e., 0.3, 0.5, 1.0, 2.0, and 3.5 wt.%. A Carver compression molder (Carver Inc., Wabash, IN, USA) was utilized to make circular samples (25 mm diameter, 0.5 mm thickness) at 210 °C under 38 MPa pressure for 10 min for morphological, electrical, and rheological characterizations.

### 2.2. Materials Characterization

#### 2.2.1. Catalyst and N-MWNT Characterization

Raman Spectroscopy: Raman spectroscopy was employed to investigate the structural defects of N-MWNTs. Ten different spots of N-MWNTs powders were recorded with a WITec alpha 300 R Confocal Raman Microscope (WITec GmbH, Ulm, Germany) with laser radiation of 532 nm, 10× objective, and a laser power of 24 mW, integration time of 50 s.

Thermogravimetric analysis (TGA): the carbon purity and the thermal stability of N-MWNTs were probed using a Thermogravimetric Analyzer (TA Instruments, Model: Q500, New Castle, DE, USA). The samples were heated under air atmosphere (Praxair AI INDK) from room temperature to 900 °C at a rate of 10 °C/min. The samples were kept at 950 °C for 10 min before cooling down to room temperature.

X-ray photoelectron spectroscopy (XPS) analysis: XPS spectra of powdered N-MWNTs samples were recorded on a Thermofisher Scientific K-Alpha XPS spectrometer (Thermofisher Scientific, E. Grinstead, UK). Monochromatic Al K_α_ X-ray with a nominal spot size diameter of 400 µm was used for the characterization. Charge compensation was applied using the combined e^−^/Ar^+^ flood gun connected to the instrument. For each sample, a survey spectrum was recorded with a low energy resolution (pass energy–150 eV), where only C, O, and N elements were identified. Then, for all C1s, O1s, and N1s regions, high resolution (pass energy-25 eV) spectra were acquired. Relative atomic % was obtained from these peaks using the sensitivity factors supplied with the instrument (C1s–1; O1s–2.881; N1s–1.676). Peak fitting was accomplished on these regions on the peaks with a Lorentzian/Gaussian mix of 30%. Symmetric peak shapes were implemented with the exclusion of the main peak, which has been assigned to sp^2^-C. All instrument operation and data processing were carried out with the Avantage v. 5.962 software supplied with the instrument.

Transmission electron microscopy (TEM): employing transmission electron microscopy (TEM) (FEI, Hillsboro, OR, USA), the morphology of synthesized N-MWNTs was evaluated. To prepare the samples for TEM, about 1.0 mg of the N-MWNTs powders was suspended in 10 mL ethanol, then bath sonicated for 15 min. A drop of this suspension was positioned on the carbon side of a standard TEM grid enclosed with a ~40 nm holey carbon film (EMS, Hatfield, PA, USA). N-MWNTs lengths and diameters measurements for statistical analysis were performed for more than 120 individual N-MWNTs using ImageJ software.

#### 2.2.2. Nanocomposites Morphology and Structure

Light microscopy (LM): the micro dispersion state of N-MWNTs within the PVDF matrix was quantified using light microscopy (LM) in transmission mode on thin cuts (5 µm thickness) of the compression-molded samples. The samples were cut with a Leica microtome RM2265 (Leica Microsystems GmbH, Wetzlar, Germany) equipped with a diamond knife. An Olympus microscope BH2 equipped with a CCD camera DP71 (both from Olympus Deutschland GmbH, Hamburg, Germany) was employed to take images from different cut sections (15 cuts, area of each: 600 × 800 µm^2^).

Transmission electron microscopy (TEM): ultrathin sections of the samples (60 nm thickness) were cut utilizing an ultra microtome EM UC6/FC6 (Leica, Austria) setup with an ultrasonic diamond knife at ambient temperature. Transmission electron microscopy (TEM) of the ultra microtomed cuts was performed using TEM LIBRA 120 (Carl Zeiss SMT, Oberkochen, Germany) with an acceleration voltage of 120 kV.

Rheology: employing a rheometer (Anton-Paar MCR 302, Graz, Austria), the rheological behavior of the nanocomposites was studied. All the rheological measurements were performed at the nanocomposites processing temperature (i.e., 210 °C), using a parallel-plate geometry with a diameter of 25 mm and a constant gap-size of 0.3 mm.

Broadband electrical conductivity: the broadband electrical conductivity of the nanocomposites was measured with a Bio-Logic Impedance Analyzer SP-200 EIS (BioLogic, Seyssinet-Pariset, France) in the frequency range of 10^−1^ to 10^6^ Hz. The impedance analyzer was connected to a sample holder (Solartron 12962, West Sussex, UK) with an electrode diameter of 10 mm. The amplitude of the applied voltage was 100 mV (Vrms~70 mV). Prior to the measurements, the electrodes were painted on the samples using silver paste.

## 3. Results and Discussion

### 3.1. Characterization of N-MWNTs

#### 3.1.1. Transmission Electron Microscopy

**Figure 1:**
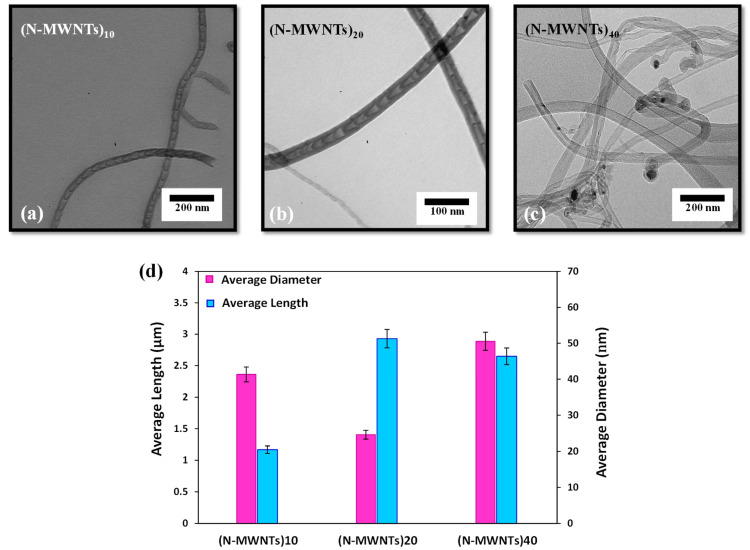
(**a**–**c**) TEM images of N-MWNTs grown over different ratios of catalyst, (**d**) average diameter and length of N-MWNTs synthesized over different catalysts.

TEM images were used to observe the morphology and graphitic structure of the N-MWNTs synthesized using different ratios of catalyst to support (C/S). Figure 1a–c show that changes in the ratio of C/S play a dominant role in the final morphology of the N-MWNTs. As shown in Figure 1a–c, N-MWNTs synthesized over 40 wt.% of iron catalyst, (N-MWNTs)_40_,are mostly open-channel, while N-MWNTs grown on 10 wt.% and 20 wt.% of iron catalyst, namely (N-MWNTs)_10_ and (N-MWNTs)_20_, respectively, have bamboo-like configurations. It is also clearly observed that the N-MWNTs with bamboo-like structure have a thicker wall in the straight region than in the disordered region, which results in more irregularities, imperfections, and roughness in the structure of the bamboo-like N-MWNTs. The presence of such defective structures in the synthesized N-MWNTs could be attributed to the substitution of nitrogen atoms by carbon atoms in the hexagonal graphitic-based structure of CNTs [29,35,36,37].

Figure 1d demonstrates the average diameter and length of N-MWNTs synthesized over different ratios of C/S. As obtained from statistical analysis, the synthesized N-MWNTs presented a range of size that was achieved by measuring the length and diameter of more than 100 individual N-MWNTs. The results shown in Figure 1d represent the average values. It is worth noting that the length and diameter of N-MWNTs are of critical importance in electrical applications of conductive filler/polymer nanocomposites; i.e., longer N-MWNTs could more readily provide conductive filler/polymer nanocomposites with superior electrical performance [38,39,40].

Comparing the lengths of the N-MWNTs synthesized over different C/S ratios, it is observed that all N-MWNTs, except for (N-MWNTs)_10_ which has the lowest length, had almost the same average length of about 2.8 µm. The smaller length of (N-MWNTs)_10_ could be attributed to the lower amount of the active catalyst. There is not any consistent trend in terms of increasing diameter and length in N-MWNTs; however, there is a consistent trend in the overall growth of the N-MWNTs. Increasing Fe concentration provides more active catalyst sites. In (N-MWNT)_10_, longitudinal growth was not eye catching while lateral growth was significant. (N-MWNT)_20_ followed the opposite trend, longitudinal growth was much higher than lateral growth. However, for (N-MWNT)_40_, both longitudinal and lateral growth were considerable. Therefore, increasing Fe concentration led to higher growth, although it appears differently in each concentration. In 10 wt% Fe, it emerges by lateral growth, at 20 wt% it appears by longitudinal growth, and at 40 wt% Fe it leads to both longitudinal and lateral growth.

#### 3.1.2. X-ray Photoelectron Spectroscopy

**Figure 2:**
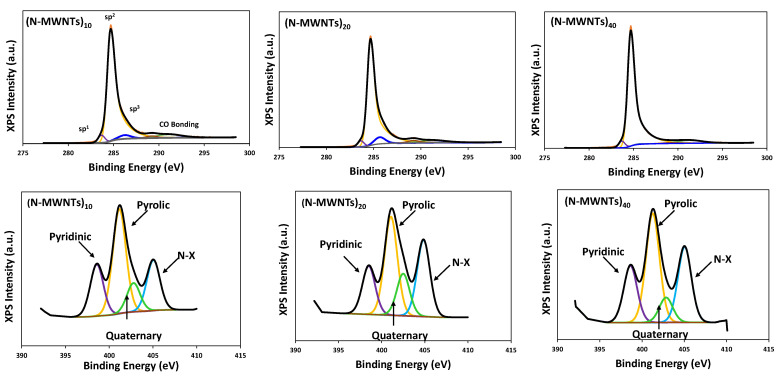
XPS Spectra and C1s and N1s of N-doped CNTs synthesized using different ratios of catalyst.

**Figure 3:**
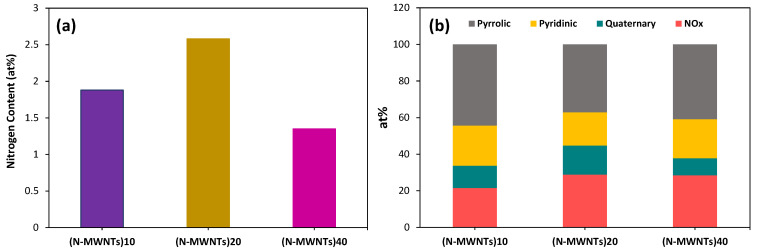
(**a**) Plot of nitrogen content in structure of N-MWNTs as a function of catalyst loadings to alumina support, (**b**) plot of the percentage of different types of nitrogen incorporation in N-MWNTs for different growth catalyst.

One of the most important factors that affects the morphological and electrical properties of the nanotubes is the amount of nitrogen and type of the nitrogen bonding. Thus, in order to investigate the effect of C/S ratio on the nature and quantity of nitrogen bonding, XPS technique was employed. The high-resolution core level photoelectron peak positions and XPS survey spectra of N-MWNTs are observed in Figure 2. The N1s and C1s peaks for synthesized N-MWNTs were deconvoluted into separated peaks, as indicated in Figure 2. Peak fitting was carried out using a Lorentzian/Gaussian mix of 30%. The detailed information regarding C1s and N1s peaks from the XPS spectra is tabulated in Table 1 and Figure 3, respectively. The C1s peak, which appears at 284.67 eV, is assigned to sp^2^ carbon hybridization, while the C1s peak around 286.23 eV is related to sp^3^ hybridization of carbon atoms in hexagonal graphitic-based structure of the nanotubes. As is extensively reported in the literature [29], graphitic materials, such as CNTs, contain carbon atoms mainly in sp^2^ or sp^3^ hybridization. The higher the content of sp^2^ hybridization of CNTs, the higher is their graphitization degree. For N-MWNTs synthesized in this work, all the synthesized N-MWNTs had almost the same content of sp^2^ hybridized carbon atoms (~85 at %); thus, it is concluded that doping of CNTs with nitrogen atoms did not change the sp^2^ hybridization of carbon atoms significantly.

To further analyze the elemental structure of N-MWNTs, the ratio of the area of N1s and C1s peaks in XPS spectra was used to obtain the amount of nitrogen content, see Figure 3a. The results indicate that the atomic percentage (at%) of nitrogen in synthesized N-MWNTs grown over 20 wt.% of iron catalyst is 2.58 at%, while (N-MWNTs)_10_ and (N-MWNTs)_40_ have considerably lower amounts of nitrogen content, equal to 1.38 and 1.35 at%, respectively.

As already reported in our previous work and the other literature [26,41,42,43], different types of bonding are created between nitrogen atoms within the basic hexagonal structure of CNTs; including graphitic, pyridinic, and pyrrolic. For creating graphitic nitrogen, three sp^2^ orbitals of nitrogen take part in forming covalent bonds with the adjacent carbon atoms such that the remaining valence electrons occupy the p_z_ orbitals of nitrogen. Pyridinic nitrogen atoms are sp^2^ hybridized, where two of its five valence electrons are confined to lone-pairs and do not become incorporated in the conjugated p system. Pyrrolic nitrogen atoms are sp^3^ hybridized and are part of a five-membered ring structure. Besides the above mentioned nature of nitrogen atoms in N-MWNTs, some nitrogen oxides were also found within synthesized N-MWNTs, which are also attributed to the pyridinic nitrogen [44]. Figure 3b demonstrates that different types of nitrogen bonding were formed in the synthesized N-MWNTs, among which Pyrrolic and N-X dominate. The ratio of each nitrogen type was mainly affected by the ratio of C/S.

The contribution of the pyridinic and pyrrolic species is relatively higher for (N-MWNT)_10_ and (N-MWNT)_40_ compared to (N-MWNT)_20_, whereas the opposite behavior is observed for Quaternary species. Muñoz-Sandoval et al. [45] showed that samples containing higher concentrations of pyrrolic nitrogen formed zigzagged carbon fibers, whereas higher concentrations of quaternary nitrogen in the samples develop robust wavy and straight carbon nanotubes. The interesting point about the influence of C/S ratio on properties of N-MWNT grown by CVD is that there is no linear relationship between increasing the catalyst concentration and properties. There is an optimum for choosing the C/S ratio based on desirable properties.

#### 3.1.3. Raman Spectroscopy

**Figure 4:**
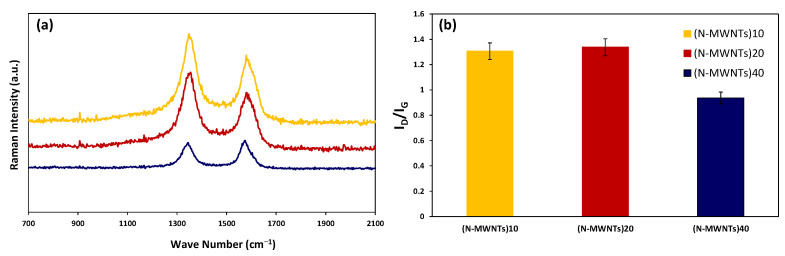
(**a**) Raman spectra of N-MWNTs synthesized using different ratios of catalyst. (**b**) Intensity ratio of D- and G-band (I_D_/I_G_) as a function of different synthesized N-MWNTs.

To investigate the graphitization and crystallization degree of the synthesized N-MWNTs, Raman spectroscopy was utilized as a powerful and non-destructive technique for the characterization of N-MWNT powdered samples. The G band, arising from the stretching of C–C bonding in graphitic materials, which is common to all sp^2^ carbon forms, appeared around ~1600 cm^−1^. The D band, which shows defect-active mode and appears around ~1360 cm^−1^, indicates the presence of structural defects, such as pentagon–heptagon pairs, vacancies, heteroatoms, and impurities. Accordingly, the ratio of D band to G band intensities (I_D_/I_G_) is implemented as a tool in Raman spectroscopy to evaluate the impact of C/S ratio on the structural imperfections and doping of N-MWNTs. Figure 4 depicts the I_D_/I_G_ ratios of N-MWNTs synthesized over the different ratio of iron catalyst to alumina support. N-MWNTs synthesized over 20 wt.% of iron catalyst show the highest I_D_/I_G_ ratio indicating the most defective structure for this sample.

In previous studies, it is claimed that there is a direct relationship between nitrogen content in N-MWNTs and I_D_/I_G_ ratio [28]. This is in good agreement with our observation showing that a higher I_D_/I_G_ results in less graphitized structures for N-MWNTs. The high intensity of I_D_/I_G_ is attributed to the presence of amorphous carbon on the exterior walls of CNTs, or the availability of oxygen and nitrogen atoms within the symmetric hexagonal structure of CNTs that impairs their graphitization and electronic properties.

#### 3.1.4. Thermogravimetric Analysis (TGA)

**Figure 5:**
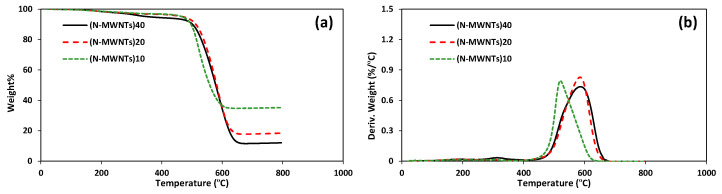
(**a**) TGA, (**b**) DTA plot of N-MWNTs grown over different ratios of Fe catalyst.

Thermogravimetric analysis (TGA) and differential thermal analysis (DTA) of the synthesized N-MWNTs deliver insightful information about their thermal stability and synthesis yield. Figure 5 shows that the inflection points for N-MWNTs synthesized over 10, 20, and 40 wt.% Fe catalyst are 480 °C, 505 °C, and 503 °C, respectively. It is expected that the thermal resistivity in air will be inversely proportional to the nitrogen content and the presence of amorphous carbon in the N-MWNTs [19,33,45], which applies well to (N-MWNTs)_10_ and (N-MWNTs)_40_. In the case of (N-MWNTs)_20_, the number of defects and crystallinity obtained from TEM, Raman (I_D_/I_G_), and XPS (nitrogen content) results are not in line with thermal stability. This suggests that parameters other than structural defects control the thermal stability of the N-MWNTs [33,46].

It is also observed that the remaining residues, i.e., alumina substrate and iron catalyst particles, for (N-MWNTs)_10_, (N-MWNTs)_20_, and (N-MWNTs)_40_, were 35%, 18.1%, and 11.8%, respectively. According to the inverse relationship between carbon purity and residual mass, it can be concluded that the carbon purity scales with the Fe catalyst concentration. Furthermore, only a single peak is detected in the DTA curves for all N-MWNTs, demonstrating that all N-MWNTs were composed of one phase [47]. To recapitulate, (N-MWNTs)_40_ possesses the highest synthesis yield and optimum thermal stability, signifying the synthesis of highly crystalline N-MWNTs at this C/S ratio. Therefore, better final electrical properties for its nanocomposites are expected.

### 3.2. Characterization of N-MWNTs/PVDF Nanocomposites

#### 3.2.1. Morphology of N-MWNTs/PVDF Nanocomposites

Ultimate functional properties of polymer-based nanocomposites are greatly governed by various factors, such as intrinsic properties of the polymer matrix and nanofillers along with the dispersion quality of the nanofillers. It has been well established that the electrical properties of nanocomposites can be enhanced by generating interconnected 3D networks of the conductive fillers in the polymer matrices achieved by a proper dispersion state. In general, the network formation of a conductive filler at a smaller fraction of the filler is highly appreciated. This can be achieved by breaking the primary agglomerates of N-MWNTs into the individual N-MWNTs without decreasing the aspect ratio. Therefore, dispersion quality of nanofillers (in this case N-MWNTs) was assessed over different length scales using imaging techniques.

**Figure 6:**
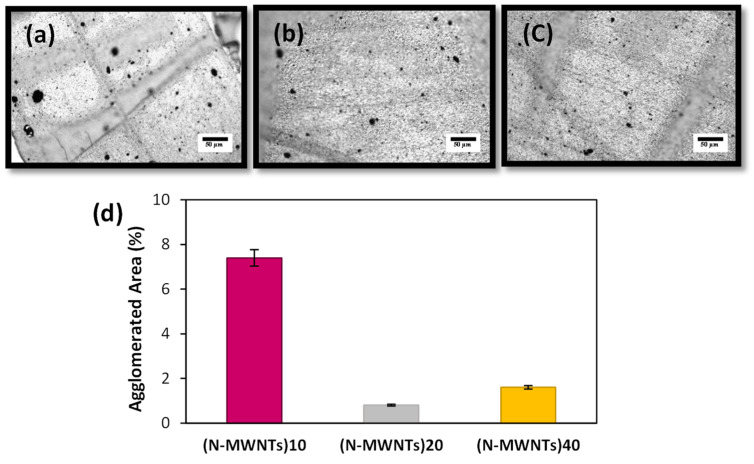
LM images of microtomed N-MWNTs/PVDF nanocomposites with 2.0 wt.% N-MWNTs synthesized at various catalyst concentration, (**a**) 10 wt.%, (**b**) 20 wt.%, (**c**) 40 wt.%, (**d**) Agglomerate area ratio of thin cuts of 2.0 wt.% N-MWNTs/PVDF nanocomposites.

First, the dispersion of N-MWNTs and the presence of primary agglomerates of N-MWNTs in PVDF (N-MWNTs/PVDF) in micron scale were analyzed using optical microscopy. The quantitative analysis was performed by estimating the average agglomerate area ratio. Figure 6a–c represent optical micrographs of PVDF composites containing 2 wt.% N-MWNTs synthesized with various C/S ratios. The effect of catalyst amount on the microscopic dispersion is evident. The nanocomposites containing N-MWNTs synthesized with the higher amount of Fe catalyst manifest better dispersion quality in PVDF. For instance, PVDF nanocomposites containing 2 wt.% of N-MWNTs synthesized with 40 wt.% Fe catalyst depict 1.6% of agglomerated area, followed by 0.8% and 7.4% for 20 wt.% and 10 wt.% of Fe catalyst, respectively. The low dispersion quality of (N-MWNTs)_10_/PVDF stems from the inferior purity of (N-MWNTs)_10_. 

**Figure 7:**
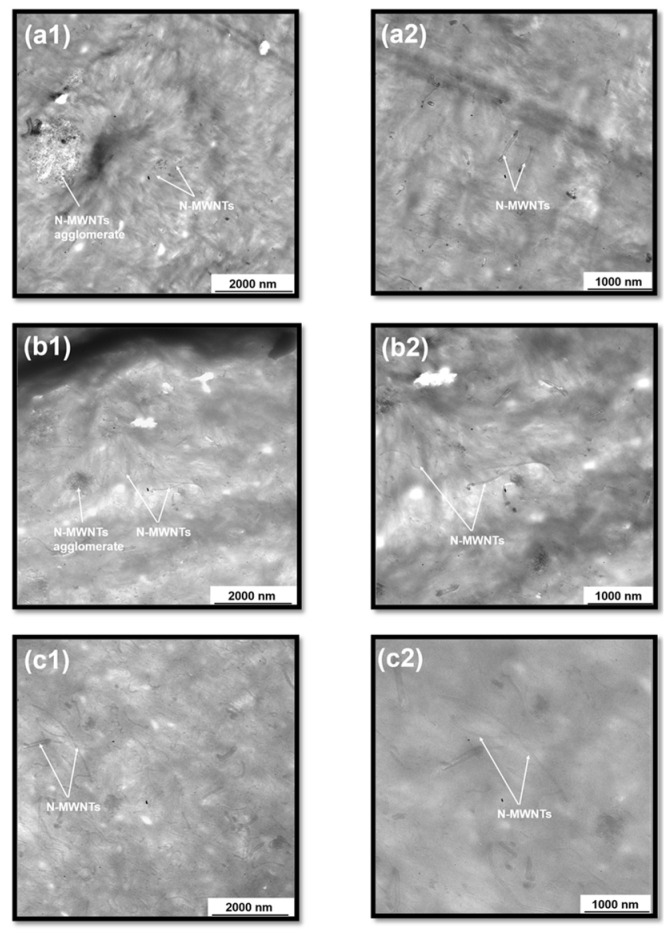
TEM micrographs of PVDF nanocomposites containing 2 wt.% N-MWNTs synthesized with Fe catalyst of (**a1**,**a2**) 10 wt.%, (**b1**,**b2**) 20 wt.%, and (**c1**,**c2**) 40 wt.%.

The nanoscopic dispersion of N-MWNTs was studied using TEM micrographs. Figure 7 shows TEM micrographs of PVDF nanocomposites containing 2 wt.% N-MWNTs. Huge agglomeration for (N-MWNTs)_10_/PVDF is also obvious in nanoscopic scale (Figure 7(a1)). At the given amount of (N-MWNTs)_10_/PVDF, no interconnected networks were realized. High magnification TEM micrographs depict few individual N-MWNTs dispersed in the PVDF. It is important to note that, at a given concentration of N-MWNTs, since the purity of N-MWNTs synthesised using 10 wt.% Fe catalyst was low (as referred from TGA), the amount of crystalline N-MWNTs taking part in the formation of interconnected networks in the PVDF matrix is also relatively low. In addition, the aspect ratio of N-MWNTs synthesised using 10 wt.% Fe catalyst was relatively lower compared to 20 and 40 wt.% (as referred from TEM analysis on nanofillers). This reveals its disability in forming 3D interconnected networks. Besides, the highly defective structure of (N-MWNTs)_10_ (confirmed by Raman spectroscopy) acts as a stress concentration point, leading to breakage of N-MWNTs during nanocomposite processing. This further lead to a poor ability of (N-MWNTs)_10_ to develop conductive networks in PVDF matrix, resulting in a high percolation threshold in PVDF nanocomposites, which will be discussed later.

The degree of network formation of N-MWNTs is significantly enhanced in higher Fe catalyst concentrations. That is, an enhanced dispersion quality at the nanoscopic scale is observed with an increase in the amount of Fe catalyst (Figure 7(b1,b2,c1,c2)). Only a few small agglomerates of N-MWNTs synthesized with 20 wt.% and 40 wt.% catalyst. This further leads to an enhanced ability to form an interconnected network structure in PVDF.

#### 3.2.2. Rheology of N-MWNTs/PVDF Nanocomposites

**Figure 8:**
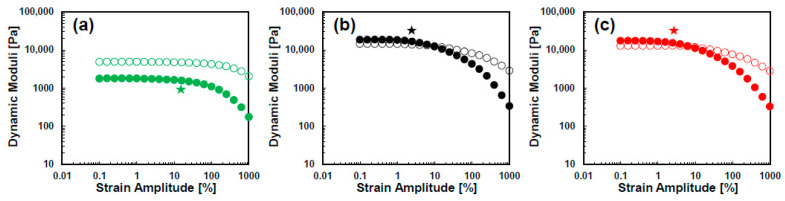
Oscillatory amplitude sweep response of N-MWNTs/PVDF nanocomposites containing 3.0 wt.% of carbon nanotubes synthesized on different catalyst concentrations for strain amplitudes of $\gamma_{0}$ = 0.1–1000% at an angular frequency of ω= 1 rad/s using parallel-plate geometry at a gap-size of 0.3 mm at 210 °C. Open symbols represent loss modulus (*G*″) and solid symbols represent storage modulus (*G*′). (**a**) PVDF/N-MWNTs synthesized with 10 wt.% Fe, (**b**) PVDF/N-MWNTs synthesized with 20 wt.% Fe, and (**c**) PVDF/N-MWNTs synthesized with 40 wt.% Fe. The star symbols show the critical strain amplitude (linear to nonlinear transition).

In this section, rheology is assisted as a strong tool to grant further insight into the dispersion state and network structure of the nanocomposites. An oscillatory strain sweep test in which the samples are subjected to an increasing strain amplitude at a controlled frequency was used to investigate the microstructure of the systems. Figure 8 shows the strain sweep results for N-MWNTs/PVDF nanocomposites containing 3.0 wt.% nanotubes (strain amplitude range is $\gamma_{0}$ = 0.1 to 1000% at a locked angular frequency of ω = 1 rad/s). Based on these results (see Figure 8), a strain amplitude of $\gamma_{0}$ = 1% is small enough for our polymer nanocomposites to be in the linear viscoelastic region for the frequency-sweep test.

The transition from linear viscoelastic to nonlinear viscoelastic region takes place at the critical strain amplitude (star symbols in Figure 8), characterized by the rapid decrease in storage modulus (*G*′) value. The reduction in *G*′ is a direct consequence of nanofillers network break up and filler-matrix slippage, leading to a decrease in the number of load-bearing junctions [48,49,50,51,52]. Moreover, it was shown that, for a strong-linked network, the critical strain amplitude decreases to lower values by the introduction of more nanofillers into the polymer media [4,52,53]. This is due to the higher sensitivity of the rigid 3D network structure of the nanofillers to the input deformation (i.e., strain amplitude) [53,54].

As can be seen in Figure 8, comparing the viscoelastic responses of the samples reveals significant differences in their network structure. For PVDF nanocomposites containing (N-MWNTs)_20_ and (N-MWNTs)_40_, *G*′ is greater than *G*″ in the small amplitude region. This solid-like behavior (*G*′ > *G*″) signifies the presence of a percolated network and the ability of these N-MWNTs to form an integrated 3D network [55]. This initial solid-like behavior undergoes a solid-to-liquid-like transition (*G*′ < *G*″) by increasing the amplitude of deformation. However, for PVDF containing (N-MWNTs)_10_, *G*′ is less than *G*″ in all the probed strain amplitude windows and no solid-like behavior was observed, which is suggestive of weakness of this N-MWNT-type nanofiller to form a percolated rheological network even at high concentrations. Moreover, the critical strain amplitude is smaller for the nanocomposites with (N-MWNTs)_20_ and (N-MWNTs)_40_ compared to nanocomposites containing (N-MWNTs)_10_ (see the star symbols in Figure 8). This can be explained in accordance with the formation of a more developed (rigid) network as a result of a higher number of load-bearing junctions in N-MWNTs synthesized with 20 or 40 wt.% catalyst. Hence, these rigid networks are more likely to break down at smaller strain amplitudes.

**Figure 9:**
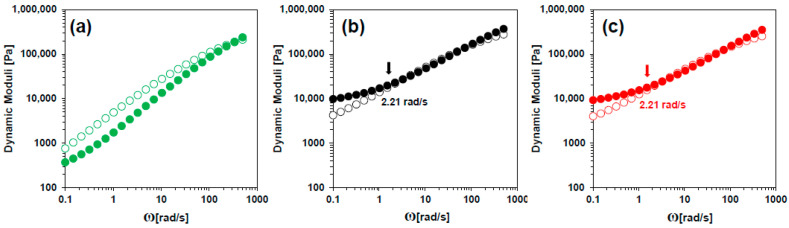
Loss modulus (*G*″) (open symbols) and storage modulus (*G*′) (solid symbols) of N-MWNTs/PVDF nanocomposites containing 3.0 wt.% of carbon nanotubes synthesized on different catalyst concentrations for strain amplitudes of 1% using parallel-plate geometry at a gap-size of 0.3 mm at 210 °C. (**a**) PVDF/N-MWNTs synthesized with 10 wt.% Fe, (**b**) PVDF/N-MWNTs synthesized with 20 wt.% Fe, and (**c**) PVDF/N-MWNTs synthesized with 40 wt.% Fe. Arrows represent the location of second crossover points (*G*″ = *G*′).

The results of the small amplitude ($\gamma_{0}$ = 1%) frequency-sweep tests on the N-MWNT-based polymer nanocomposites are shown in Figure 9. In this test, the frequency was swept from 500 to 0.1 rad/s. In line with the strain amplitude test results, *G*′ is smaller than *G*″ for (N-MWNTs)_10_/PVDF and no evidence of network formation can be observed. On the contrary, the viscoelastic behavior of the other N-MWNT-type polymer nanocomposites is noticeably different. *G*′ is greater than *G*″ at low frequencies and a distinct nonterminal behavior is observable for PVDF nanocomposites with 3.0 wt.% (N-MWNTs)_20_ and (N-MWNTs)_40_. Additionally, there is a secondary crossover at the low frequency of 2.21 rad/s for these samples. The first crossover occurs at higher frequency. The first crossover originates from the nature of polymer segmental motions, which is suggestive of typical elastic response in extremely short time scales.

However, the secondary crossover at low and intermediate frequencies (longer relaxation times) is a sign of a transition from a viscous response to a solid-like response. This solid-like behavior for PVDF nanocomposites with (N-MWNTs)_20_ and (N-MWNTs)_40_ confirms formation of the nanofillers’ network structure in these samples. As already shown in the TEM analysis of the nanofillers, the length of the N-MWNTs synthesized with 20 and 40 wt.% Fe is almost the same. Thus, the same rheological behavior of these two N-MWNTs originates from their roughly similar physical features.

Based on the above discussion, it can be concluded that PVDF nanocomposites containing (N-MWNTs)_20_ and (N-MWNTs)_40_ are capable of formation of a robust 3D network, verified by an apparent solid-like response at long time scales. However, PVDF nanocomposites containing (N-MWNTs)_10_ could not form a percolated network. Further insight into the network structure of the nanocomposites is provided by the intra-cycle viscoelastic response of the samples in the next section.

#### 3.2.3. Lissajous-Bowditch Plots

**Figure 10:**
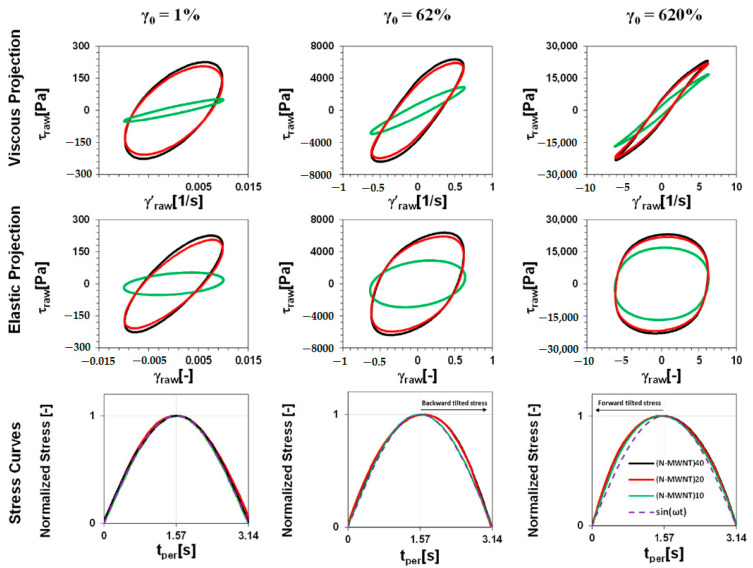
Lissajous–Bowditch loops of N-MWNTs/PVDF nanocomposites containing 2.0 wt.% of N-MWNTs synthesized at various Fe catalyst concentrations, 10 wt.% (green), 20 wt.% (red), and 40 wt.% (black) using parallel-plate geometry (gap size of 0.3 mm) at 210 °C. Elastic and viscous projections and stress curves are presented at strain amplitudes of 0 = 1.0, 62, and 620%, and an angular frequency of ω = 0.1 rad/s.

Intra-cycle nonlinear viscoelastic characterization can provide insightful information regarding nanocomposites’ structural features [52,56,57,58,59,60]. In this section, we utilized the stress decomposition method to interpret the intra-cycle viscoelastic responses of our nanocomposites to further elucidate their microstructure. In this method, nonlinear stress response of the materials is decomposed into elastic and viscous contributions. Beyond the linear viscoelastic region, due to the excitation of higher harmonics in the output shear stress waveform, stress response is not a perfect sinusoid. Therefore, by tracing the distortion of the intra-cycle output stress waveform, useful information about the microstructural features can be obtained [25,60,61]. A useful way of representing the intra-cycle output stress waveform is in the form of Lissajous–Bowditch (LB) loops. In LB plots, the time varying stress σ(t) is plotted as a function of the oscillating strain γ (t) = $\gamma_{0}$ sin *ω*t (elastic projection), or as a function of the instantaneous shear rate $\dot{\gamma }$ (t) = $\gamma_{0}$*ω*cos*ω*t (viscous projection). Figure 10 exhibits elastic projection, viscous projection, and stress curves for nanocomposites containing 2.0 wt.% of N-MWNTs at an angular frequency of 1 rad/s and various strain amplitudes ($\gamma_{0}$) of 1, 62, and 620%, each representative of small amplitude oscillatory shear (SAOS), medium amplitude oscillatory shear (MAOS), and large amplitude oscillatory shear (LAOS) regions, respectively.

In the linear region, the stress response of a purely elastic material is in phase with the deformation and its stress response is out of phase with the shear rate by π/2. Thus, in LB plots, for purely elastic material, it is expected to see a line in elastic projection and a circle in viscous projection. On the contrary, for purely viscous material, a circle and a line would appear in the elastic and viscous projections, respectively. Therefore, for viscoelastic materials such as polymer nanocomposites, ellipsoidal patterns are expected in LB plots in the linear viscoelastic region (LVR) [48]. Any distortion from elliptical shape in the LB plots verifies the emergence of nonlinearity in materials’ viscoelastic behavior [54].

As can be seen in Figure 10, in the SAOS region ($\gamma_{0}$ = 1%), the LB loops in both viscous and elastic projections for all nanocomposites are elliptical, indicating linear viscoelastic responses. However, the slope of the major axis of the elastic LB loop of (N-MWNTs)_10_/PVDF in this region is significantly smaller compared to its other counterparts. This behavior indicates a more liquid-like response in the viscoelastic character of the (N-MWNTs)_10_/PVDF, which can be correlated to the inability of the (N-MWNTs)_10_ to bridge and construct a 3D network structure. In addition, in this region, the narrower LB loops in the elastic projection compared to viscous projection for (N-MWNTs)_20_/PVDF and (N-MWNTs)_40_/PVDF verifies their elastic-dominant response. By increasing the strain amplitude, the viscous LB loops become noticeably narrower, whereas elastic LB loops become broader. This behavior, conjugated with the transition of LB loops from elliptical shape to non- elliptical shape for all nanocomposites, signifies entering the nonlinear region upon rupture of the nanofillers’ network [54]. For instance, the emergence of intra-cycle nonlinearity in the LAOS region ($\gamma_{0}$ = 620%) leads to distortion of the elastic LB plots to a curvilinear rectangle trajectory for all nanocomposites. In this region, the dramatic differences between (N-MWNTs)_10_/PVDF and the other two nanocomposites diminish due to complete collapse of the N-MWNTs network structure under the applied deformation, thereby actuating the viscoelastic response of the systems with viscoelastic behavior of the pristine PVDF matrix [52].

We also investigated the shape of the stress waveform (normalized periodic stress waveforms against time plotted in Figure 10) to provide complementary information regarding the network structure. As can be seen in the stress curves in Figure 10, in the LVR region ($\gamma_{0}=$1%), the stress output waveform is sinusoidal for all nanocomposites, indicating a linear viscoelastic response. Transition from SAOS to MAOS region for (N-MWNTs)_20_/PVDF and (N-MWNTs)_40_/PVDF samples can be discerned by the emergence of a “backward titled” stress curve, which is good evidence of the excitation of the higher harmonics in the stress waveform. However, (N-MWNTs)_10_/PVDF still follows a sinusoidal periodic stress waveform in the MAOS region. A transition from “backward titled” to “forward titled” is observable for (N-MWNTs)_20_/PVDF and (N-MWNTs)_40_/PVDF by going from the MAOS to the LAOS region. In the LAOS region, the stress output waveform of PVDF/(N-MWNTs)_10_ also became distorted and deviated from a sinusoidal wave and shows a “forward titled” behavior [61,62]. Thus, the intracycle nonlinear behavior at sufficiently large strain amplitudes ($\gamma_{0}$ = 620%) becomes apparent as “forward titled” stress waveform for all nanocomposites. Taken together, no evident difference can be discerned between the LB loops of (N-MWNTs)_20_/PVDF and (N-MWNTs)_40_/PVDF samples in neither LB plots nor stress curves. These results are in good agreement with the data of the frequency and strain sweep tests.

#### 3.2.4. Broadband Electrical Conductivity of N-MWNTs/PVDF Nanocomposites

Broadband electric conductivity represents nomadic charge transfer in the material under the applied external electric field. The broadband electrical conductivity is depicted as $\sigma =\sigma_{DC}+A{\left(w\right)}^{s}$. In this case, $\sigma_{DC}$ represents frequency-independent DC conductivity. This corresponds to the transfer of nomadic charges in phase with the applied electric field. On the other hand, $A{\left(w\right)}^{s}$ represents frequency-dependent AC electrical conductivity generated due to the out-of-phase transfer of nomadic charges. The electrical properties of polymer-based nanocomposites are governed by the inherent electrical and structural properties, and network formation of the conductive nanofillers (in this case N-MWNTs) in a given polymer matrix. In the case of insulative polymer matrices, the incorporation of a conductive filler facilitates transport of charges through the interconnected networks of the fillers. In this context, the degree of network formation is controlled by the concentration, aspect ratio, and dispersion quality of the fillers. Therefore, the electrical conductivity can be also used as a strong tool to study the network formation of these conductive fillers in the bulk of a polymer matrix.

**Figure 11:**
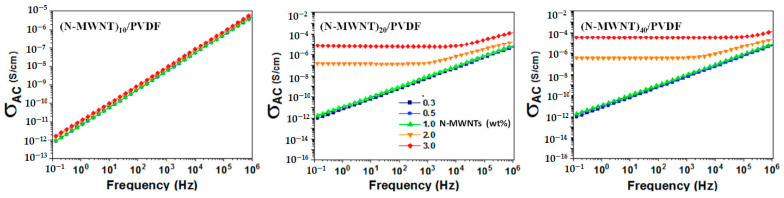
AC electrical conductivity as a function of frequency for PVDF nanocomposites containing N-MWNTs synthesized with catalyst concentration of Fe catalyst.

Figure 11 shows AC electrical conductivity as a function of frequency for PVDF nanocomposites containing various amounts of the synthesized N-MWNTs. It has been well established that, at a critical concentration, nanocomposites depict a dramatic increase in the electrical conductivity. This is mainly due to the generation of the 3D network of the conductive fillers in a given polymer matrix. This is often referred to as the electrical percolation threshold. Above the percolation threshold, further increase in the amount of the conductive fillers leads to the further establishment of the conductive network, which results in numerous least resistive pathways for nomadic charge transfer. It is also observed that, at a very high amount of conductive filler, with increasing the loading of the filler, although the networks of the conductive filler are further developed, the electrical conductivity does not change. This is because the ultimate conductivity of the nanocomposites is limited by the contact resistance between discrete conductive fillers.

As can be seen in Figure 11, for the PVDF nanocomposites containing (N-MWNTs)_10_, the conductivity increases with the frequency, signalling an insulative behavior. An insulative behavior, at a given amount of the N-MWNTs, suggests the absence of an electrically conductive network. Hence, conduction, tunnelling, or hopping of the nomadic charges is unlikely. This can be assigned to various factors disturbing the conductive networks of (N-MWNTs)_10_ in PVDF matrix such as poor dispersion quality, which was confirmed by rheological responses and TEM images of the nanocomposite, along with the highly distorted structure of N-MWNTs. Moreover, the carbon purity of the N-MWNTs also controls the formation of the conductive networks. As we observed from TGA analysis, the (N-MWNTs)_10_ depicted the lowest carbon purity. Hence, the small amount of the graphitic carbon in the (N-MWNTs)_10_-incorporated nanocomposites is insufficient to facilitate the nomadic charge transfer. This further results in an enhanced resistance for charge transfer through (N-MWNTs)_10_, leading to low electrical conductivity. Moreover, the poor dispersion quality of (N-MWNTs)_10_ in PVDF matrix also leads to a decrease in effective aspect ratio. Hence, a small number of N-MWNTs are able to take part in developing conductive networks in PVDF matrix, resulting in an inferior electrical conductivity at a given concentration of (N-MWNTs)_10_.

The electrical conductivity is enhanced remarkably for PVDF nanocomposites containing N-MWNTs synthesized with a higher fraction of Fe catalyst. The nanocomposites containing N-MWNTs synthesized with 20 and 40 wt.% Fe catalyst depict a percolation threshold between 1 to 2 wt.%, where a frequency-independent conductivity is observed at low frequencies. At percolation threshold, nanocomposites represent semi conductive nature. That is, further increase in the concentration of N-MWNTs (i.e., above 2.0 wt.%) leads to an enhanced electrical conductivity along with extended frequency independent plateau. Moreover, the electrical conductivity scaled with the amount of Fe catalyst used for synthesis of N-MWNTs. Thus, the highest electrical conductivity, at any given amount of N-MWNTs, is observed for nanocomposites containing N-MWNTs synthesized using 40 wt.% catalyst. This suggests the highest ability of (N-MWNTs)_40_ to develop conductive networks in the polymer matrix.

In summary, although the rheological properties and dispersion quality of (N-MWNTs)_20_/PVDF and (N-MWNTs)_40_/PVDF nanocomposites are almost the same, samples containing (N-MWNTs)_40_ demonstrate slightly higher electrical properties. This could be attributed to higher intrinsic conductivity of (N-MWNTs)_40_ due to factors such as lower nitrogen content, less defects, and more graphitized structure corroborated by the XPS and Raman spectroscopy results. Taken together, the altered structural and electrical properties of N-MWNTs and, more importantly, the dispersion quality of N-MWNTs in the polymeric matrix significantly affected the extent of network formation of N-MWNTs in PVDF and the ultimate charge transport properties of nanocomposites.

## 4. Conclusions

The effects of several parameters such as temperature, catalyst, and time of synthesis on physical and chemical structural features of carbon nanotubes (CNTs) in the chemical vapor deposition method have been widely studied. However, the influence of the amount of catalyst during synthesis has been underestimated. Hence, in this work, we showcased the remarkable effect of iron (Fe) catalyst to substrate ratio on the physical and chemical properties of nitrogen-doped carbon nanotubes (N-MWNTs). To achieve this, N-MWNTs were synthesized at three catalyst to substrate ratios, i.e., 10, 20, and 40 wt.% iron. Employing TEM, XPS, Raman spectroscopy, and TGA techniques, it was revealed that the catalyst to substrate ratio has a significant impact on the diameter, length, crystallinity, carbon purity, nitrogen content, and nitrogen-bonding type of the synthesized N-MWNTs. The TEM results showed that lateral growth was higher than longitudinal growth for N-MWNTs synthesized with 10% Fe catalyst, thereby having the lowest aspect ratio.

The nanotubes were then melt-mixed with a polyvinylidene fluoride (PVDF) to form nanocomposites. N-MWNTs synthesized with 10% Fe catalyst (N-MWNTs)_10_ showed the worst dispersion quality in the PVDF compared to its counterparts as verified by optical microscopy and TEM images. Linear and nonlinear rheological measurements conducted on the nanocomposites confirmed the trend of the dispersion quality observed by imaging techniques. To obtain further insight into the microstructure of the N-MWNT-based polymer nanocomposites, Lissajous–Bowditch loops were studied; these loops showed that (N-MWNTs)_10_ were not able to form a 3D network structure. Thus, (N-MWNTs)_10_/PVDF nanocomposites exhibited an insulative behavior even at a high concentration (3.0 wt.%) of (N-MWNTs)_10_, and this was corroborated by AC conductivity measurements. However, increasing Fe catalyst to 20% and 40% resulted in percolated nanocomposites with good dispersion states and provided a low rheological and electrical percolation threshold. Although TEM and rheological results showed that (N-MWNTs)_20_ and (N-MWNTs)_40_ had similar abilities to establish a continuous network of N-MWNTs within the PVDF matrix, (N-MWNTs)_40_/PVDF nanocomposites showed higher electrical properties. The better electrical properties of (N-MWNTs)_40_/PVDF nanocomposites are attributed to the intrinsic chemical features of (N-MWNTs)_40_ such as nitrogen content and nitrogen-bonding type. In summary, the catalyst to substrate ratio has a dramatic impact on the final physical and chemical structure of nanomaterials synthesized via the CVD method. Hence, this catalyst to substrate ratio must be considered during catalyst preparation.

## Figures and Tables

**Table 1:** XPS peak positions data for C1s line of N-MWNTs synthesized using different ratios of iron catalyst to alumina support.

Sample Type	Component Assignment	Peak BE (eV)	Atomic Conc. (%)
**(N-CNTs)_10_**	sp^1^-C	283.67	3.76
	sp^2^-C	284.67	81.11
	sp^3^-C	286.23	1.85
	CO Bondings	291.05 289.22	10.13
**(N-CNTs)_20_**	sp^1^-C	283.67	3.23
	sp^2^-C	284.67	83.26
	sp^3^-C	286.23	5.94
	CO Bondings	291.05 289.22	7.75
**(N-CNTs)_40_**	sp^1^-C	283.67	3.37
	sp^2^-C	284.67	89.01
	sp^3^-C	286.23	0.55
	CO Bondings *	291.05 289.22	7.07

## Data Availability

The dataset generated and/or analyzed during the current study are available from the corresponding author on reasonable request.
